# Control of CXCR2 activity through its ubiquitination on K327 residue

**DOI:** 10.1186/s12860-014-0038-0

**Published:** 2014-10-22

**Authors:** Héloise M Leclair, Sonia M Dubois, Sandy Azzi, Julie Dwyer, Nicolas Bidère, Julie Gavard

**Affiliations:** 1 CNRS, UMR8104, 22 rue Mechain, Paris, 75014, France; 2 INSERM, U1016, 22 rue Mechain, Paris, 75014, France; 3 Universite Paris Descartes, Sorbonne Paris Cite, 12 rue de l’Ecole de Medecine, Paris, 75006, France; 4 Institut Cochin, 22 rue Mechain, Rm. 306, Paris, 75014, France

**Keywords:** Chemokine receptor, Ubiquitination, Arrestin, Internalization, Signaling

## Abstract

**Background:**

The interleukin-8 chemokine (IL-8) G-protein coupled receptor CXCR2 governs pro-inflammatory and pro-angiogenic responses in leukocytes and endothelial cells. At a molecular standpoint, CXCR2 is widely reported to operate through calcium flux, phosphoinoisitide 3 kinase (PI3K) and mitogen-activated protein kinase (MAPK). While CXCR2 trafficking is suspected to be intertwined with its signaling, the exact mechanism is not fully elucidated.

**Results:**

Here, we identified the lysine 327 within the CXCR2 C-terminal tail as a key residue for ubiquitination, internalization, and signaling. First, the substitution to an arginine of K327 mutation was associated with a reduction in CXCR2 poly-ubiquitination. While WT CXCR2 was rapidly internalized following IL-8 administration, K327R mutant remained at the plasma membrane. Finally, K327R mutant failed to promote the recruitment of β-arrestin2, as estimated by imagery and bioluminescence resonance transfer. As a consequence, the activation of intracellular signaling, including both early events such as ERK phosphorylation and the increase in calcium flux, and the latter activation of the AP1 and NF-κB transcription factors, was blunted.

**Conclusions:**

Overall, our results demonstrate that CXCR2 ubiquitination on K327 residue modulates agonist-activated CXCR2 cell sorting and intracellular signaling. Thus, the inhibition of K327 ubiquitination might emerge as an effective mean to curb exacerbated CXCR2 signaling in several pathological conditions, such as inflammatory diseases and cancer.

## 1 Background

CXCR2 is a seven transmembrane G-protein coupled receptor (GPCR) for the ELR + CXCL8 (IL-8) chemokine that transduces pro-angiogenic and pro-inflammatory responses in endothelial and immune cells [1]–[5]. Indeed, IL-8/CXCR2 signaling axis plays multiple functions in the course of physiological and pathological neo-vessel formation. Likewise, IL-8 signaling is instrumental in leukocyte migration [6]. Notably, it does so by stimulating endothelial cell proliferation, permeability, and migration, and by attracting lymphocytes, macrophages, and neutrophils to perivascular regions. For instance, we recently demonstrated that IL-8 operates through CXCR2 and phosphoinositide 3-kinase γ (PI3Kγ) to promote angiogenesis and macrophage accumulation in retina, while curbing the endothelial barrier function [3]. Importantly, interfering with this pathway quelled laser-induced vascular dysfunctions in mouse retina. Similar observations were done in the context of cancers, including malignant brain tumors, prostate tumors, pancreatic ductal adenocarcinoma, chemically induced-skin tumors, Ras-mediated tumors and inflammation-driven tumors [2],[7]–[10]. These data place CXCR2 as a promising pharmacological target in many human diseases and pathological conditions [4]. In that view, targeting GPCR constitutes the primary option for pharmacological intervention and drug development [11]. However, many of these compounds act as direct antagonists leaving uncertain their ability to target pathways that are aberrantly and/or constitutively active.

Physiologically, GPCR signaling can be either twisted or interrupted by intracellular cascades implying endocytosis and ubiquitination [12]. Similarly to the CXCL12 (also known as stromal derived factor 1α, SDF1α) chemokine receptor CXCR4, ligand-activated CXCR2 undergoes endocytosis in clathrin-coated vesicles [13],[14]. In that view, the viral CXCR2 homolog vGPCR from the Kaposi Sarcoma Herpes Virus, harbors a consensus adaptin 2 (AP2) binding motif in its C-terminal tail, which drives its constitutive shuttling in clathrin-coated vesicles, and further adjusts its signaling activity [15]. Moreover, CXCR2 internalization might involve scaffold proteins, including β-arrestins, AP2, actin-binding proteins and yet to be determined PDZ ligand motif binding proteins [13],[14],[16]–[19]. However, how exactly CXCR2 signaling, intracellular sorting and ubiquitination are coordinated is not fully elucidated. This prompted us to explore the molecular contribution of lysine residues, operating as ubiquitin acceptors, to CXCR2 activity.

## 2 Results and discussion

**Figure 1:**
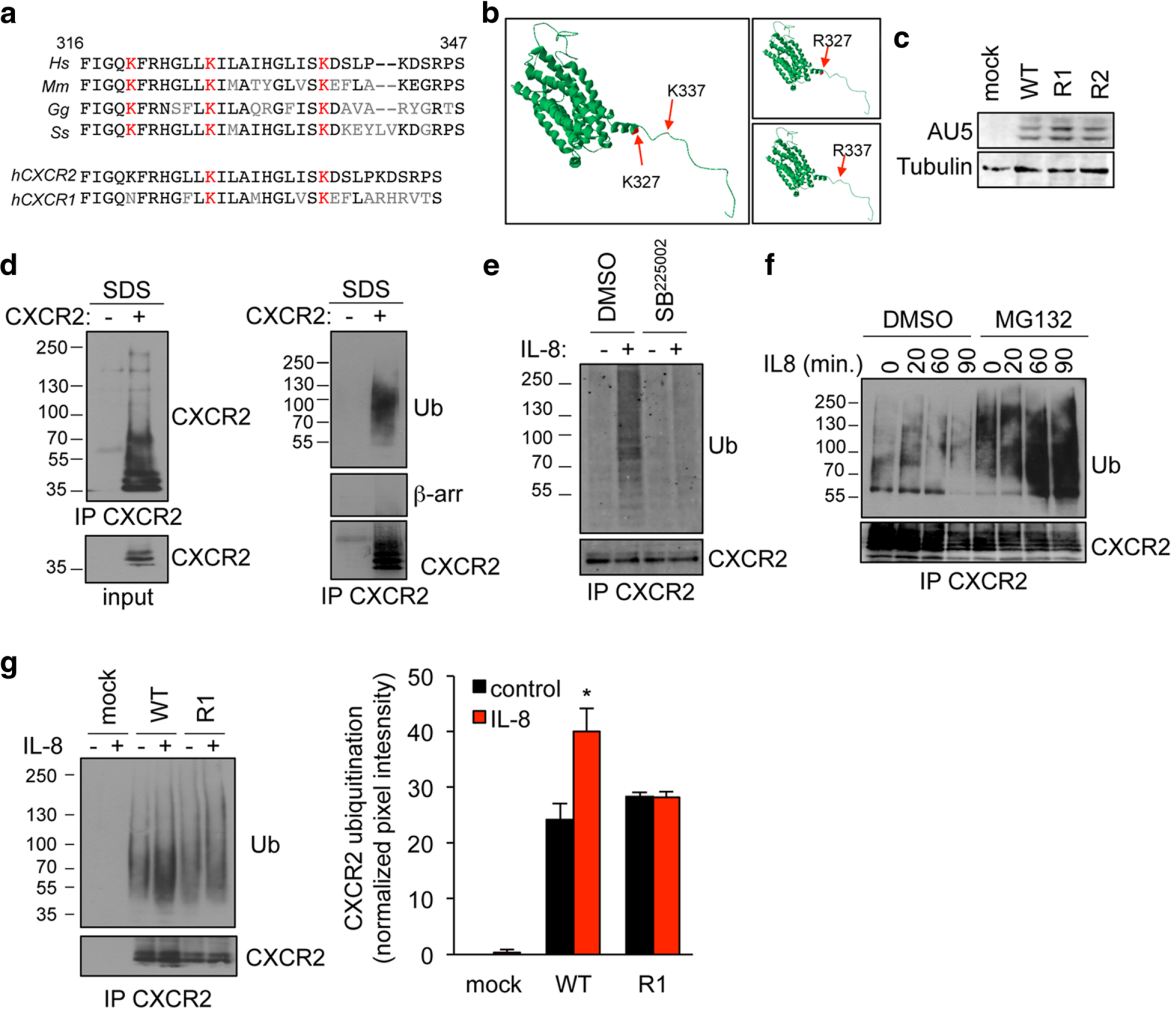
**K327 residue is critical for IL8-induced CXCR2 ubiquitination. a**. Partial sequence alignment between Homo sapiens (h), Mus musculus, Gallus gallus, and Sus scrofa CXCR2 (ILRB), and between hCXCR2 and hCXCR1 (ILRA). Conserved residues are in black, not conserved one are in grey. Conserved lysines are highlighted in red. **b**. CXCR2 secondary structure was predicted using LOMETS software and modeled using Swiss-PDB viewer. K327 and K337 are indicated with an arrow and colored in red. **c-g**. HEK-293T cells were transfected with pCEFL-AU5 plasmid (mock), in frame wild type (WT), K327R (R1), and K337R (R2) mutant forms of FL CXCR2. **c**. Anti-AU5 and anti-Tubulin western-blots were performed 24 h later. **d**. CXCR2 immunoprecipitation (IP) was performed onto SDS denaturated protein lysates from IL-8-treated samples (15 min, 50 ng/ml), and further analyzed by western-blots for CXCR2, β-arrestin and CXCR2. Input for CXCR2 is also presented. **e-f**. Alternatively, cells were pre-incubated with CXCR2 antagonist (SB225002, 50 μM, 1 hour) or MG132 proteasome inhibitor (25 μM, 45 min). **g**. CXCR2 IP and WB were performed as in (d) in starved cells (-) and in IL-8-challenged cells (+, 15 min, 50 ng/ml). Pixel intensities of the ubiquitination lanes were quantified using Image J software and normalized to untreated mock lane. Means + sem are shown. Each panel is representative of three independent experiments. *p < 0.05.

The ligation of G-protein coupled receptor CXCR2 drives its relocation into endosomal compartments, where it could be targeted for lysosomal degradation [13]. This dynamic process has been shown to rely on specific motifs, including lysine residues within the C-terminal domain [13]. To investigate which lysine could contribute to the fine-tuning of CXCR2 trafficking, we first proceed to amino-acid sequence alignment between residues 316 and 347 on the human sequence. Our *in silico* analysis identified two lysine residues on position 327 and 337 in the human sequence, conserved in pig, mouse, and chicken, and shared between CXCR2 and CXCR1 (Figure 1a). As expected, structural prediction did not reveal major changes in the overall CXCR2 molecular organization in the context of K-R mutations (Figure 1b). Point mutation in each of these two lysines was thus engineered on the full-length (FL) receptor, harboring an AU5 tag. The expression of K327R (named R1) and K337R (named R2) FL CXCR2 mutants was found similar to the one of wild type (WT) when transfected in HEK-293T cells (Figure 1c). These constructs have been thus used throughout the study. Because lysine residues functions as ubiquitin-acceptors, we next setup the experimental conditions to visualize CXCR2 ubiquitination, using validated methods for intracellular targets [20],[21]. CXCR2 immunoprecipitated fractions were analyzed under denaturing conditions, *ie* 1% SDS final concentration to prevent non-covalent protein interactions but without heat denaturation that could favor membrane protein aggregation. Using these conditions, the monoclonal anti-ubiquitin antibody (clone P4D1) readily detected modified CXCR2 in the high molecular-weight fraction (Figure 1d). Additionally, denaturation prior immunoprecipitation dissociated putative contaminations with associated ubiquitinated partners of CXCR2, such as β-arrestins (Figure 1d). Similarly, ubiquitination addition was detected in the CXCR2 immunoprecipitated fractions from endothelial cells that endogenously expressed the receptor (Additional file 1: Figure S1). As a further control, CXCR2 antagonist SB225002 [3],[22] was sufficient to curtail CXCR2 ubiquitination in response to IL-8 (Figure 1e). Of note, CXCR2 ubiquitination species accumulated in cells pretreated with the proteasome inhibitor MG132, suggesting that ubiquitination marks CXCR2 for degradation (Figure 1f). We next examine the ubiquitination profile of K-R CXCR2 mutants. Interestingly, the typical ubiquitination ladder discerned in response to IL-8 challenge in WT CXCR2-expressing HEK-293T cells was profoundly reduced when K327 residue was substituted for an arginine (Figure 1g). Our data thus suggest that the K327 residue is critical for K-linked ubiquitination of activated CXCR2.

**Figure 2:**
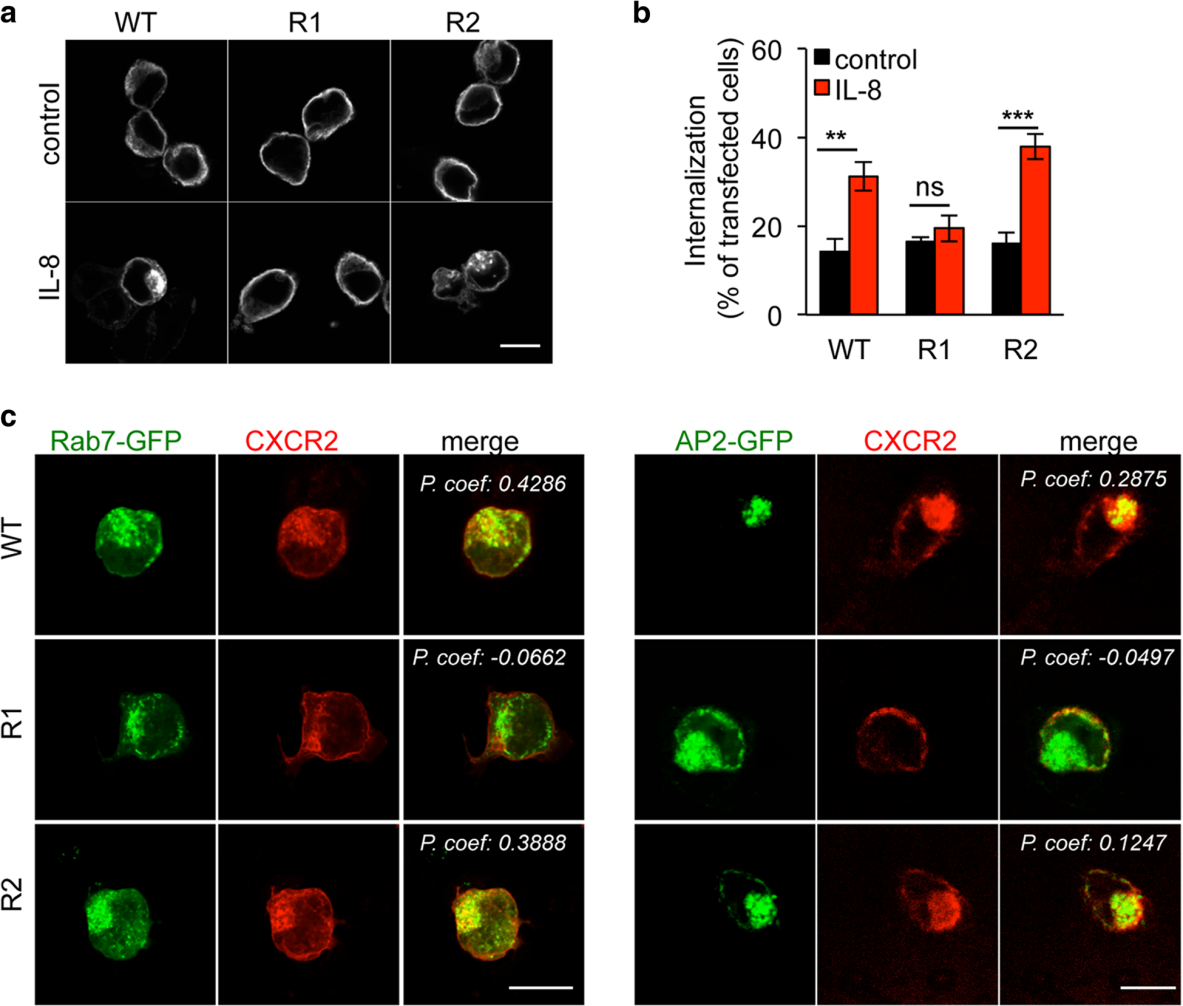
**K327 residue drives CXCR2 trafficking in response to IL-8.** HEK-293T cells were transfected with pCEFL-AU5 plasmid (mock), containing in frame wild type (WT), K327R (R1), and K337R (R2) mutant forms of FL CXCR2. **a**. CXCR2 staining was analyzed by confocal microscopy in 1 h-starved cells (control) followed by IL-8 stimulation (50 ng/ml, 15 min). **b**. CXCR2 antibody uptake was performed as described in methods and analyzed by confocal microscopy in control and IL-8 stimulated cells. Internalization was estimated by the percentage of cells with CXCR2 vesicular staining per transfected cells. Results are expressed as the mean ± sem, n = 150 cells. **c**. Alternatively, IL-8-challenged cells were co-transfected with either Rab7-GFP or AP2-GFP 24 h prior confocal analysis. Pearson’s correlation coefficient (P. coef) was calculated on 5 and 3 samples for Rab7-GFP and AP2-GFP, respectively. Each panel is representative of three independent experiments. Scale bars: 10 μm. **p < 0.01, ***p < 0.001.

We next aimed at understanding how K327 could modulate CXCR2 trafficking. In this scenario, localization of K-R CXCR2 mutants was further characterized by confocal microscopy (Figure 2). In starved non-stimulated HEK-293T cells, WT CXCR2 primarily labeled plasma membrane and redistributed in internal vesicle-like dots shortly following IL-8 challenge (15 minutes, Figure 2a). By contrast, K327R CXCR2 remained accumulated at the plasma membrane regardless of IL-8 treatment. On the other hand, the K337R mutant mimicked WT CXCR2, as it shuttled from the plasma membrane to internal vesicles (Figure 2a). This was found similar to endogenous CXCR2 dynamics in IL-8-challenged THP1 monocytes (Additional file 2: Figure S2). Likewise, CXCR2 internalization, as monitored through antibody uptake assay followed by an acid wash, was enhanced in response to IL-8 administration, unless K327 was mutated (Figure 2b). Co-labeling with Rab7 and AP2, which illuminates late and early endosomes, respectively, indeed supports the notion that WT and K337R CXCR2 were endocytosed upon IL-8 stimulation (Figure 2c). Conversely, plasma membrane staining of the K327R mutant was not curbed by IL-8 treatment, unlike WT and K337R mutant (Figure 2c). Altogether, our results show that the K327R point mutation is sufficient to restrain CXCR2 internalization upon ligand binding, suggesting therefore that this residue is critical to ensure proper trafficking of the activated receptor.

**Figure 3:**
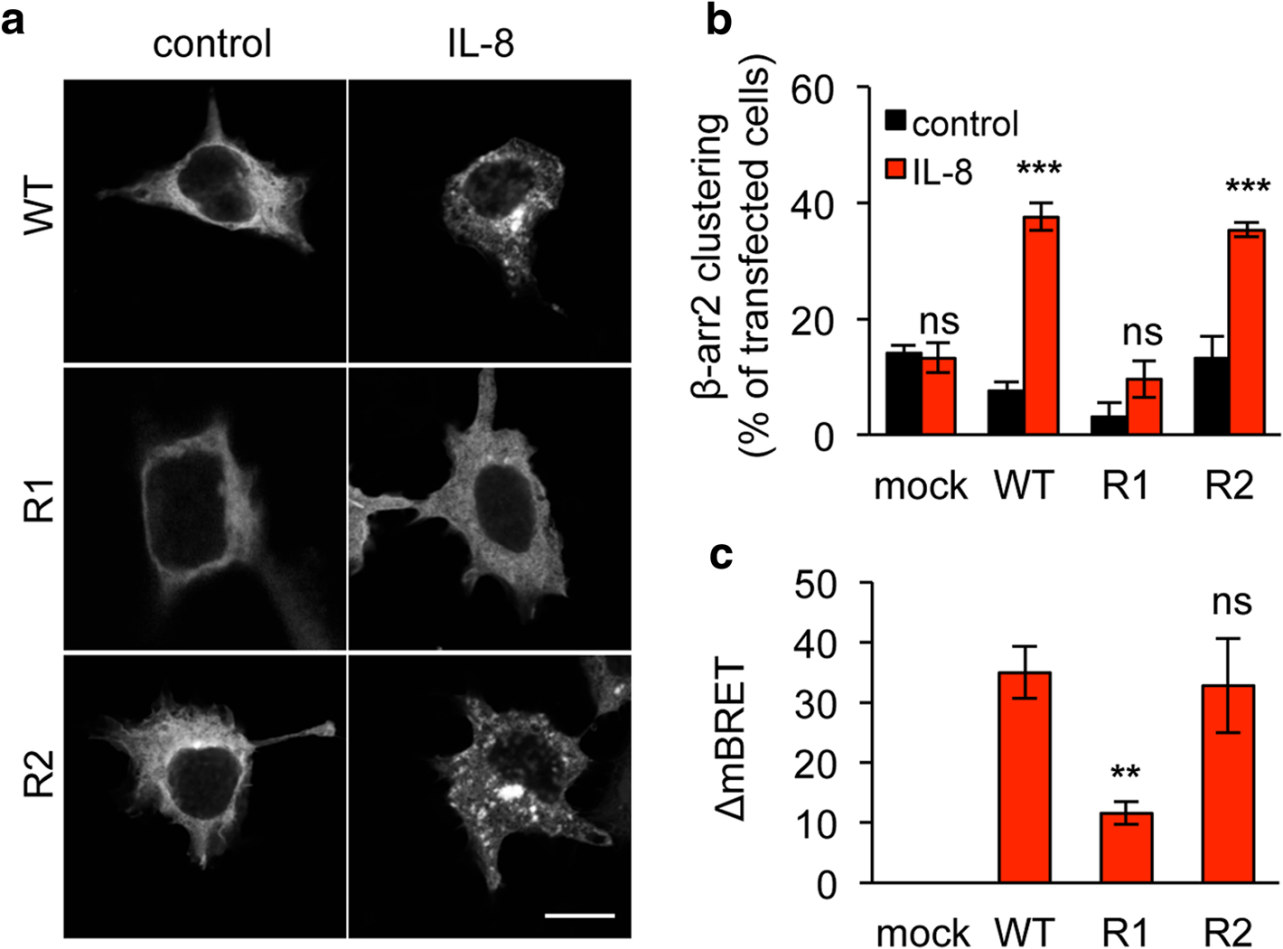
**K327R mutation eliminates β-arrestin2 recruitment.** HEK-293T cells were transfected with pCEFL-AU5 plasmid (mock), containing in frame wild type (WT), K327R (R1), and K337R (R2) mutant forms of FL CXCR2. One day later, cells were starved (control) or exposed to IL-8 (50 ng/ml, 15 min). **a-b**. Cells were additionally transfected with β-arrestin2-GFP (βarr2) and the percentage of cells with clustered β-arrestin2-GFP staining was evaluated. Results are expressed as the mean ± sem, n = 150 cells. **c**. BRET analysis was performed as described in methods, ΔmBRET was calculated and the mean ± sem is shown. Each panel is representative of three independent experiments. Scale bars: 10 μm. **p < 0.01, ***p < 0.001.

**Figure 4:**
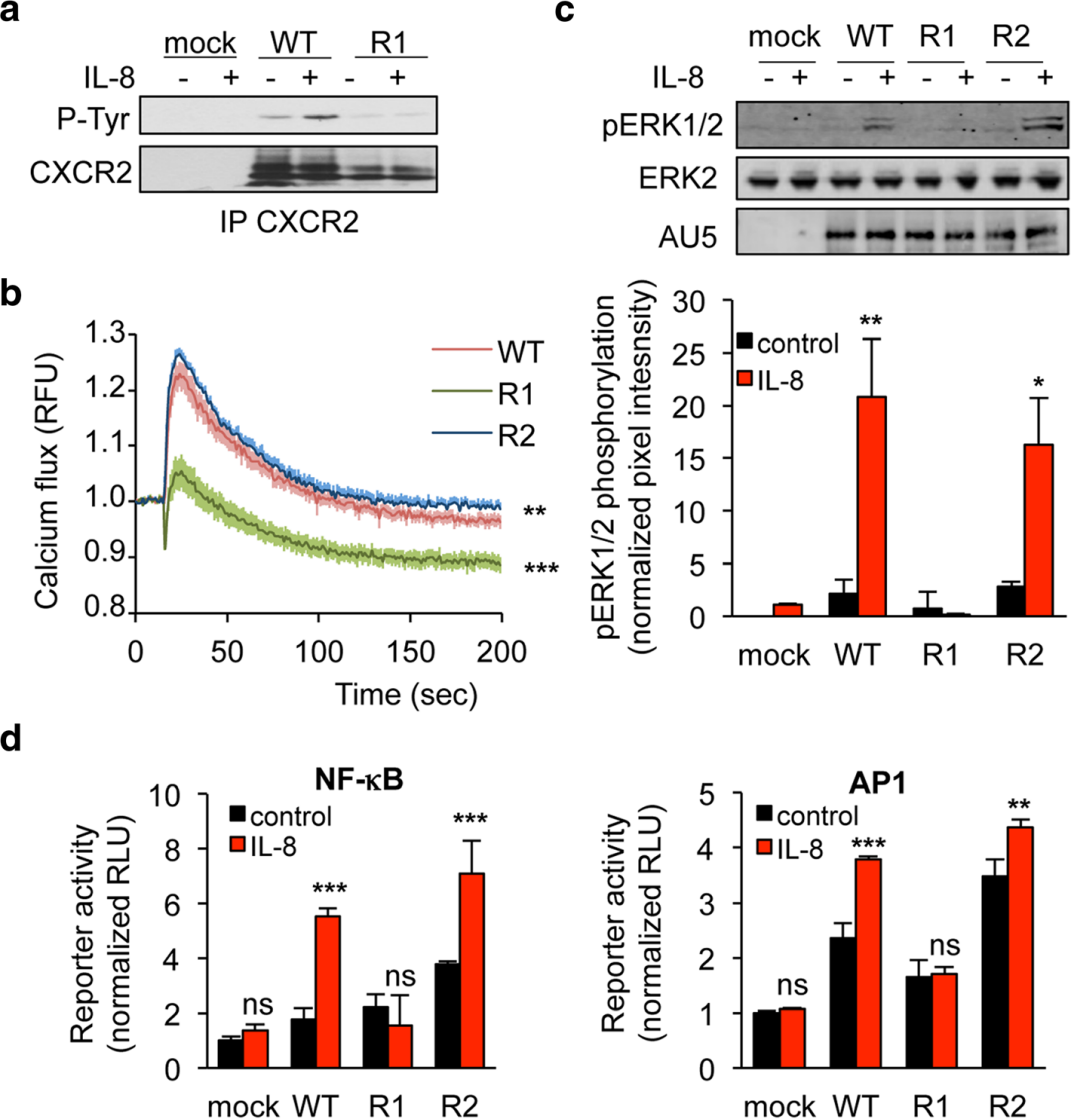
**K327R mutation abrogates CXCR2 signaling in response to IL-8.** HEK-293T cells were transfected with pCEFL-AU5 plasmid (mock), containing in frame wild type (WT), K327R (R1), and K337R (R2) mutant forms of FL CXCR2. One day later, cells were starved (control) or exposed to IL-8 (50 ng/ml, 15 min, unless specified). **a**. Anti-phospho-Tyr (4G10), anti-CXCR2 western-blots were performed in CXCR2 immunoprecipitated (IP) fraction. **b**. Calcium flux was measured using the Fluo4NW probe in response to IL-8 for the indicated times. Fluorescent units were normalized to control conditions. **c**. Anti-AU5, anti-pERK1/2 and anti-ERK2 western-blots were performed. Pixel intensities of the pERK1/2 lanes were quantified using Image J software and normalized to total ERK2. **d**. Promoter activity of both NF-κB and AP1 was monitored through luciferase-based assays. Each panel is representative of three independent experiments. Means + sem are shown. **p < 0.01, ***p < 0.001.

We next investigated whether K327 residue participates to CXCR2 signaling ability. To this aim, one of the most proximal events in GPCR activation, namely β-arrestin2 activation was first assessed. Interestingly, K327R single substitution prevented from IL-8-induced β-arrestin2 clustering, observed normally in cells transfected with WT and K337R CXCR2 (Figure 3a-b). Likewise, K327R CXCR2 mutant less efficiently activated β-arrestin2 in response to IL-8, as estimated through Bioluminescence Resonance Energy Transfer (BRET) technology (Figure 3c). As phosphorylation is also an important hallmark of GPCR activation upon ligation [23], we checked the status of CXCR2 tyrosine phosphorylation in immunoprecipitation experiments (Figure 4a). As expected, IL-8 drove WT CXCR2 phosphorylation. In sharp contrast, the signal was barely detectable when K327R mutant was ectopically expressed in HEK-293T (Figure 4a). Secondly, intracellular signaling, namely extracellular regulated kinase 1/2 (ERK1/2) phosphorylation and increase in calcium influx, was evaluated in WT, K327R and K337R CXCR2 expressing HEK-293T cells (Figure 4b-c). Our data showed that IL-8 could not elicit either CXCR2 and ERK1/2 phosphorylation, or the elevation in intracellular calcium concentration in K327R CXCR2-transfected cells. Furthermore, luciferase-based reporter assays unveiled that K327 mutation abrogates AP1 and NF-κB activation downstream of CXCR2 stimulation by IL-8 (Figure 4d). Thus, our data demonstrate that the signaling function of CXCR2 is compromised when the K327 residue is not available for ubiquitination.

## 3 Conclusions

We report here that the conserved K327 residue on CXCR2 is a key ubiquitin acceptor upon IL-8 challenge in ectopic cell models. Of note, interfering with this post-translational modification abrogates CXCR2 trafficking and signaling. In this scenario, CXCR2 poly-ubiquitination, by a yet to be determined ligase, precedes β-arrestin2 recruitment and CXCR2 internalization, and is therefore placed upstream in the IL-8-mediated intracellular signaling cascade. Our data thus highlight an original option to specifically and finely tune CXCR2 signaling in pathological situations, where aberrantly elevated, such as tumor angiogenesis and inflammation.

## 4 Methods

### 4.1 Cell culture and transfections

HEK-293T cells (ATCC) were maintained in Dulbecco’s modified Eagle’s medium supplemented with 10% fetal bovine serum and antibiotics. Previously described protocols for transfection were used [24].

### 4.2 Reagents and antibodies

Recombinant human interleukin 8 (IL-8) was purchased from Peprotech, CXCR2 antagonist SB225002 and MG132 were from Calbiochem. The following antibodies were used: mouse and rabbit anti-CXCR2, rabbit anti-ERK2, mouse anti-tubulin, mouse anti-poly-ubiquitinated chains, clone P4D1 (Santa Cruz); rabbit anti-phopho-ERK1/2, rabbit anti-β-arrestin1/2 (Cell Signaling); mouse anti-phospho-tyr (4G10 clone, Millipore), and mouse anti-AU5 (Eurogentec). AlexaFluor-conjugated antibodies were from Invitrogen.

### 4.3 DNA constructs

Full length (FL) K327R (R1) and K337R (R2) CXCR2 mutants were prepared from human wild type (WT) CXCR2, cloned in frame in pCEFL-AU5 expression vector [15]. pGFP-β-arrestin2 [24], EGFP-β2-adaptin (AP2) [25] and pYFP-Rab7 were described previously [26].

### 4.4 Structural analysis

CXCR2 protein sequence was introduced to the LOMETS software [27] and the predicted secondary structure with the lowest Z-score was chosen for modeling, using the Swiss-PDB viewer 36 (www.expasy.org/spdbv/). Mutations were introduced using the mutation tool, according to the manufacturer’s instruction.

### 4.5 Immunofluorescence

HEK-293T cells were grown onto glass coverslips, fixed (PBS-paraformaldheyde 4%), permeabilized (PBS-Triton 0.5%) and blocked (PBS-BSA 3%). Following incubations with primary and AlexaFluor-conjugated secondary antibodies for 1 h in PBS-BSA 1.5%, samples were mounted (ProLong, Invitrogen), visualized and analyzed under confocal microscopy (TCS/SP5, Leica). Pearson’s coefficient was measured using the Image J plugin on 3 to 5 samples.

### 4.6 Ubiquitination assays

HEK-293T cells were transfected with mock, CXCR2-WT or K327R (R1) cDNA plasmids. 24 hours post-transfection, cells were treated as indicated and were further lysed (25 mM Hepes pH7.4, 130 mM NaCl, 10% glycerol, 8 mM β − glycerophosphate, 0.2% Igepal, 1 mM DTT, 1 mM NaVO4, 1 mM NaF, protease inhibitors, 1% SDS and 1 μl benzonase (250 U/μl, Novagen)) on ice. After 30 minutes, SDS was diluted to reach a final concentration of 0.1% and samples were precleared with Protein G agarose (Sigma) for 30 min and then incubated with antibody to CXCR2 and Protein G agarose for 1 hour at 4°C. Ubiquitination status were analyzed by immunoblot using antibody against ubiquitin.

### 4.7 Western-blots

Protein lysates were processed for western-blots as described previously [2]. Equal amounts of protein were loaded onto 4-12% acrylamide gradient gels (NUPAGE, Invitrogen) and transferred onto PVDF membranes (Thermo Scientific) for further analysis using the infrared scanner system (LiCOR).

### 4.8 Cytosolic calcium measurements

Changes in intracellular Ca2+ levels were assessed in HEK-293T as previously described [28], using Fluo-4 NW calcium indicators (Life Technologies), following the manufacturer’s instructions. Fluorescence was read in 96-well plate format at room temperature, using appropriate settings (excitation/emission 494/516 nm, BMG microplate reader).

### 4.9 Luciferase assays

Firefly luciferase constructs downstream of NF-κB and AP1 consensus sites [2] were co-transfected with Renilla luciferase pRL-TK plasmid (Promega), together with 250 ng pCEFL-AU5-CXCR2 constructs. Following incubation with IL-8 (50 ng/ml, 6 hours), luciferase activity was analyzed as described previously [2].

### 4.10 BRET assays

HEK-293T cells were transfected with 0.1 μg per well of the DNA construct coding for double-brilliance β-arrestin2 [29], together with CXCR2 constructs. Two-days after transfection, IL-8 (50 ng/ml) and DeepBlue coelenterazine substrate (5 μM, Perkin Elmer) were added. Luminescence was measured (Mithras fluorescence-luminescence detector, Berthold) as previously described in [29]. The BRET signal was determined by calculating the ratio of the light emitted by the fluorescent acceptor and the light emitted by Luc. The values (ΔmBRET) were corrected by subtracting the background BRET signals detected in non-stimulated cells.

### 4.11 Statistical analysis

All data are expressed as mean + s.e.m from three independent experiments. One-way and two-way ANOVA tests with post hoc Tukey’s analysis were used to assess statistical significance (Prism 6.0 GraphPad Software), and *P* values are indicated on figures as follows: * for p < 0.05, ** for p < 0.01, and *** for p < 0.001.

## Competing interests

The authors declare that they have no competing interests.

## Authors’ contributions

HML: designed and executed the experiments, and interpreted the data; SMD: designed and executed the experiments, and interpreted the data; SA: designed and executed the experiments, and interpreted the data; JD: designed and executed the experiments, and interpreted the data; NB: designed and executed the experiments, and interpreted the data; JG: designed and executed the experiments, interpreted the data, and prepared the manuscript. All authors read and approved the final manuscript.

## Additional files

## Supplementary Material

Additional file 1: **Human endothelial cells were serum-starved 16 h (-) prior IL-8 challenge (+, 100 ng/ml, 15 min).** CXCR2 immunoprecipitation (IP) was performed as described in methods and IP fractions were blotted against CXCR2 and ubiquitination (Ub). Click here for file

Additional file 2: **Human THP1 monocytes were serum-starved 16 h (control) prior IL-8 challenge (+, 50 ng/ml, 15 min).** CXCR2 (Santa Cruz) and AP2 (Becton Dickinson) staining were analyzed by confocal microscopy. Scale bar: 10 μm. Click here for file
