# Supplementary figures and images for: Control of CXCR2 activity through its ubiquitination on K327 residue
Source: BMC Cell Biol. 2014 Oct 22;15:38. doi: 10.1186/s12860-014-0038-0 (PMC4209453; doi:10.1186/s12860-014-0038-0)

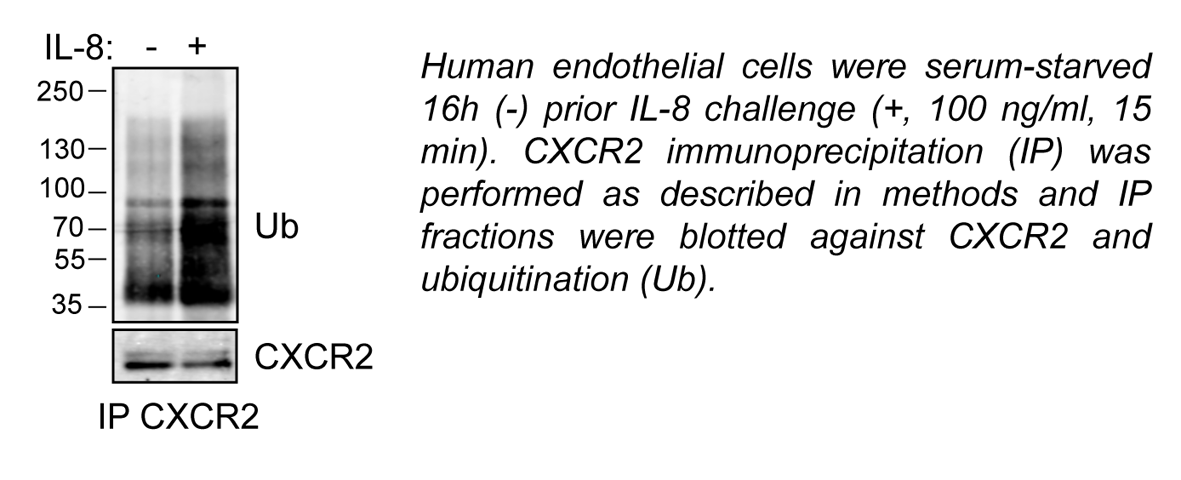

Supplement: Additional file 1: — Human endothelial cells were serum-starved 16 h (-) prior IL-8 challenge (+, 100 ng/ml, 15 min). CXCR2 immunoprecipitation (IP) was performed as described in methods and IP fractions were blotted against CXCR2 and ubiquitination (Ub). [file s12860-014-0038-0-S1.tiff]

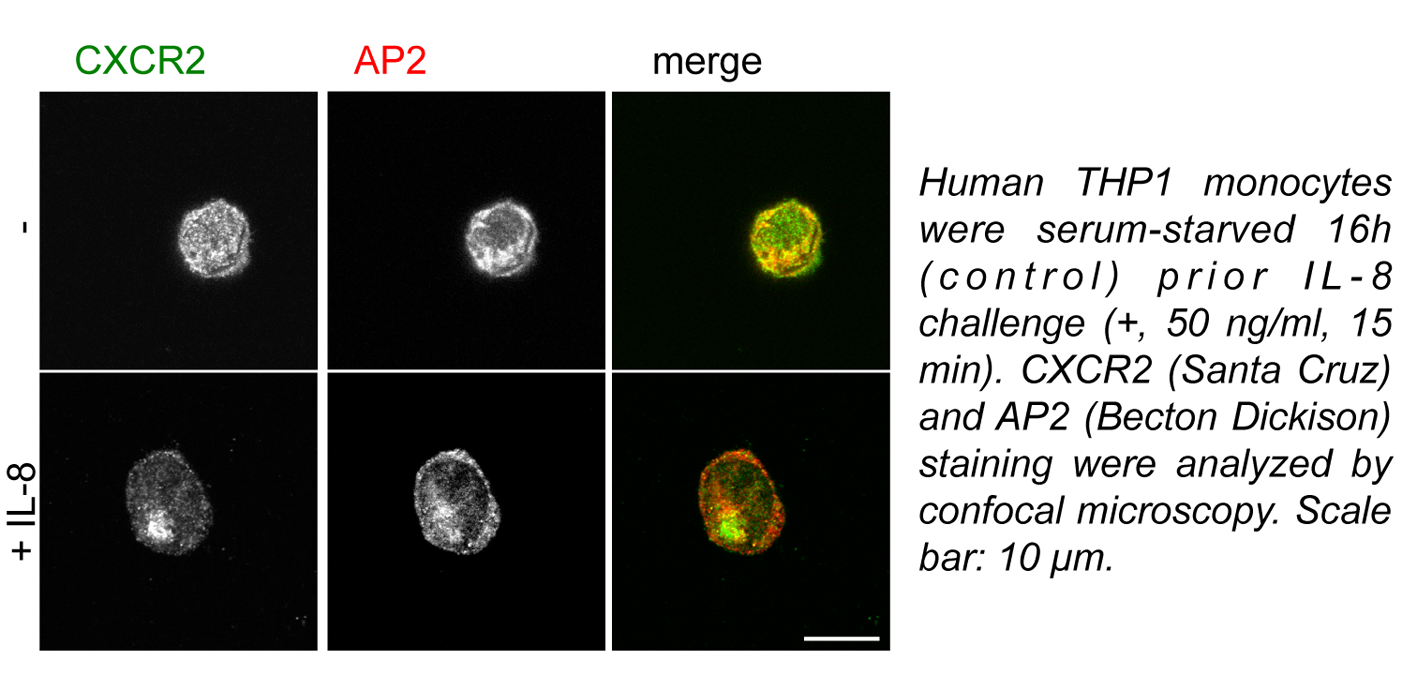

Supplement: Additional file 2: — Human THP1 monocytes were serum-starved 16 h (control) prior IL-8 challenge (+, 50 ng/ml, 15 min). CXCR2 (Santa Cruz) and AP2 (Becton Dickinson) staining were analyzed by confocal microscopy. Scale bar: 10 μm. [file s12860-014-0038-0-S2.tiff]
